# Dietary patterns and diet quality during pregnancy and low birthweight: The PRINCESA cohort

**DOI:** 10.1111/mcn.12972

**Published:** 2020-02-09

**Authors:** Monica Ancira‐Moreno, Marie S. O'Neill, Juan Ángel Rivera‐Dommarco, Carolina Batis, Sonia Rodríguez Ramírez, Brisa N. Sánchez, Marisol Castillo‐Castrejón, Felipe Vadillo‐Ortega

**Affiliations:** ^1^ UNAM School of Medicine Branch and Research Direction Instituto Nacional de Medicina Genómica México City Mexico; ^2^ Health Department Universidad Iberoamericana Mexico City Mexico; ^3^ Department of Epidemiology, School of Public Health University of Michigan Ann Arbor Michigan USA; ^4^ Centro de Investigación en Nutricion y Salud National Institute of Public Health Cuernavaca Mexico

**Keywords:** dietary patterns, low birthweight, maternal diet, maternal diet quality score, Mexico City, PRINCESA cohort

## Abstract

Although the isolated effects of several specific nutrients have been examined, little is known about the relationship between overall maternal diet during pregnancy and fetal development and growth. This study evaluates the association between maternal diet and low birthweight (LBW) in 660 pregnant women from the Pregnancy Research on Inflammation, Nutrition,& City Environment: Systematic Analyses (PRINCESA) cohort in Mexico City. Using prior day dietary intake reported at multiple prenatal visits, diet was assessed prospectively using a priori (Maternal Diet Quality Score [MDQS]) and a posteriori (dietary patterns extracted by factor analysis) approaches. The association between maternal diet and LBW was investigated by logistic regression, controlling for confounders. Adherence to recommended guidelines (higher MDQS) was associated with a reduced risk of LBW (OR, 0.22; 95% confidence interval [0.06, 0.75], *P* < .05, *N* = 49) compared with the lowest adherence category (reference group), controlling for maternal age, education, height, marital status, pre‐pregnancy body mass index, parity, energy intake, gestational weight gain, and preterm versus term birth; a posteriori dietary patterns were not associated with LBW risk. Higher adherence to MDQS was associated with a lower risk of having an LBW baby in this sample. Our results support the role of advocating a healthy overall diet, versus individual foods or nutrients, in preventing LBW.

**Key messages**

- Our findings provide evidence that higher adherence to a good quality diet during pregnancy is associated with lower risk of having a low birthweight baby.
- Characterization of dietary patterns during pregnancy using a priori and a posteriori approaches may lead to identify food groups with major contribution to diet quality and significative impact on birthweight and other perinatal outcomes.
- The present work provides valuable new knowledge to be used for improvement of pregnancy care guidelines and complementary approaches to public health.

## 1 INTRODUCTION

Low birthweight (LBW) refers to an absolute weight of <2,500 g (World Health Organization [WHO], 2004). Globally, it is estimated that 15–20% of all births are LBW infants. Low‐ and middle‐income countries account for a disproportionate burden of LBW; over 95% of the world's LBW infants are born in these countries (Cutland et al., 2017). In México, the last report of LBW rate was 8.5% in 2009 (Buekens, Canfield, Padilla, Lara Lona, & Lozano, 2013).

Restricted fetal growth and diverse influences during early development are associated with increased risk of neonatal mortality, morbidity, and altered neurodevelopment (Aarnousde‐Moens, Weisglas‐Kuperus, van Goudoever, & Oosterlaan, 2009; Jansson, 2016; Miller, Huppi, & Mallard, 2016), as well as phenotypes with increased risks for chronic disease in adulthood, including cardiovascular disease, type 2 diabetes, and hypertension. This concept, known as “fetal programming” or “developmental origins of health and disease,” has a profound impact on public health strategies for the prevention of major illnesses (Eriksson, 2016; Oestreich & Moley, 2017).

Human epidemiology and animal studies support that fetal growth is greatly influenced by maternal nutrition (Abu‐Saad & Fraser, 2010; Dimasuay, Boeuf, Powell, & Jansson, 2016; Morrison & Regnault, 2016) and is supposed to be partially mediated by changes in maternal metabolism and hormone levels (Dimasuay et al., 2016). Maternal diet composition plays a fundamental role early in pregnancy on organ development and differentiation, while in late pregnancy diet can be a major determinant of fetal growth rate and brain development (Jansson, 2016).

Individual nutrient effects on fetal growth have been studied (Brett, Ferraro, Yockell‐Lelievre, Gruslin, & Adamo, 2014; Grieger & Clifton, 2014; Kubota et al., 2013; Lager & Powell, 2012; Pannia et al., 2016), those such as iron, zinc, calcium, folate, and n‐3 polyunsaturated fatty acids, which have been associated with improved fetal health, healthier birthweight (>2,500 and <4,000 g), and increased rates of maternal and infant survival (Lowensohn, Stadler, & Naze, 2016). In addition, overall diet quality, which refers to the nutritional adequacy and food variety of an individual's dietary intake and its alignment with dietary recommendations, is also relevant. As opposed to the study of single nutrients or foods, indicators of diet quality and dietary patterns offer a broader assessment of overall adequacy of dietary intake (Borge, Aase, Brantsaeter, & Biele, 2017; Okubo et al., 2012).

Maternal diet as measured by dietary patterns as well as by diet quality scores during pregnancy has demonstrated inconsistent associations with birth outcomes. Studies carried out in New Zealand, Japan, and Denmark found that dietary patterns characterized by foods high in saturated trans fats, processed meat, sodium and added sugar, and low in vegetables, fruits, and fibre are negatively associated with birthweight (Knudsen, Orozova‐Bekkevold, Mikkelsen, Wolff, & Olsen, 2008; Okubo et al., 2012; Thompson et al., 2010). In contrast, two studies found that no particular dietary pattern was significantly associated with birthweight (Bouwland‐Both et al., 2013; Colón‐Ramos et al., 2015). Findings related with diet quality are also heterogeneous; four studies suggested that increased diet quality during pregnancy was related to a reduced risk of LBW (Chatzi et al., 2012; Emond, Karagas, Baker, & Gilbert‐Diamond, 2018; Rodriguez‐Bernal et al., 2010; Timmermans et al., 2012). On the other hand, one study found no association between Mediterranean diet (MD) or Alternative Healthy Eating Index for Pregnancy with fetal growth outcomes (Poon, Yeung, Boghossian, Albert, & Zhang, 2013).

Inconsistent results between studies may be due to the particular food context of each country and also be related to the time point on which the maternal diet is evaluated during pregnancy. Most of the previous studies describing the relationship between dietary patterns in pregnancy and fetal growth have been done in high income countries; thus, there was limited evidence demonstrating this relationship in middle‐ and low‐income populations in which diet can be a major contributor. In addition, every population adds complexity, diversity, and particular eating habits.

The aim of this study was to characterize maternal dietary patterns and diet quality during pregnancy and to evaluate the association with birthweight in a cohort of pregnant women who were clinically monitored on a monthly basis. We tested two different methods to define dietary patterns and evaluate associations with LBW, assuming diet recommendations supported by the scientific literature and adjusted to the Mexican context (Bonvecchio Arenas et al., 2015).

## 2 METHODS

### 2.1 Study design

We analysed data from a prospective cohort of pregnant women conducted in Mexico City (O'Neill et al., 2013), now known as the Pregnancy Research on Inflammation, Nutrition, & City Environment: Systematic Analyses cohort. The main purpose of the primary study was to investigate the mechanisms by which exposure to air pollutants during pregnancy could lead to perinatal complications such as preterm birth and intrauterine growth restriction.

From February 2009 to November 2014, 935 pregnant women who resided in diverse regions of metropolitan Mexico City were recruited at the Instituto Nacional de Perinatología (INPer), and public health clinics and hospitals of Mexico City's Minister of Health (SEDESA). Human subjects' approval for the study was obtained from the University of Michigan Institutional Review Board and the Ethics in Human Subjects and Research Committees of the participating Mexican institutions.

Inclusion criteria were (a) reliable recall of last menstruation; (b) agreement to prenatal visits every 4 weeks throughout their current pregnancy; and (c) written consent for their inclusion in the study. Exclusion criteria were (a) previous presence of any medical or obstetric complication in the current pregnancy and (b) presence of multiple fetuses. Women who developed pregnancy complications such as gestational diabetes and preeclampsia were referred to a specialty hospital for follow‐up. Eligibility was determined at screening and confirmed at the first visit. For the present study, an additional inclusion criterion was to have at least one complete dietary recall in both the second and third trimesters of pregnancy.

After screening for eligibility and informed consent, given at the first visit or at health clinics during recruitment, women were seen monthly over the course of their pregnancies. Information on clinical, anthropometric, and biochemical parameters and maternal diet was collected at each visit by a dedicated team composed of certified medical personnel and nutritionists with standardized training.

### 2.2 Dietary variables

Data on maternal diet were collected through a multiple‐step 24‐hr dietary recall format (24H‐DR) in the second and third trimesters of pregnancy by a nutritionist with standardized training. The multiple pass method is a five‐step approach developed by the U.S. Department of Agriculture and designed to enhance the quality of the information from the 24H‐DR (Blanton, Moshfegh, Baer, & Kretsch, 2006; Conway, Ingwersen, & Moshfegh, 2004). Dietary information in the cohort was collected as individual foods; for the present analysis, foods made of more than one ingredient (e.g., sandwich) were disaggregated into their ingredients, except for beverages, fast food, and fried snacks, which were kept as a single unit. Food portion size was calculated according to the national reference system (Perez Lizaur, Palacios Gonzalez, & Flores Galicia, 2014).

We estimated daily intake for total energy, fat, protein, carbohydrates, and fibre. In addition, daily intakes of added sugars, calcium, iron, folate, and polyunsaturated, monounsaturated and saturated fats were analysed. The estimation of daily intake of energy and nutrients was calculated by using a food composition table compiled by the Mexico's National Institute of Public Health (Instituto Nacional de Salud Pública, 2012). To estimate added sugars, we used the method proposed by Louie et al. (2015) and also used by Sanchez‐Pimienta, Batis, Lutter, and Rivera (2016) in the dietary analysis of the 2012 Mexican National Health and Nutrition Survey (Encuesta Nacional de Salud y Nutrición).

Before identification of dietary patterns, 693 individual foods were collapsed into 123 food groups to reduce complexity; these food groups were created on the basis of expected similar nutrient content. To simplify some analysis and results, we further aggregated these 123 food groups into 12 major food groups, using a grouping similar to one proposed for Mexican food items (Aburto, Pedraza, Sanchez‐Pimienta, Batis, & Rivera, 2016), also shown in Table S1.

To evaluate diet quality, we built a Maternal Diet Quality Score (MDQS) based on the Mexican Dietary Guidelines (MDG; Bonvecchio Arenas et al., 2015) and international recommendations for specific foods and nutrients. We included the following nutrients and food groups: (a) polyunsaturated fats (PUFAS), (b) added sugars, (c) fruits and vegetables, (d) red meat, (e) low fat dairy products, (f) legumes, and (g) high in saturated fat and/or added sugar (HSFAS) foods.

We used WHO intake guidelines (WHO, 2003) for fruit and vegetables (≥400 g per day) and added sugars (<10% of total energy). For PUFA, we used the Dietary Reference Intake reported by Institute of Medicine (2005; ≥6% of total energy). The recommendation in the MDG (Bonvecchio Arenas et al., 2015) for the intake of animal products is based on the World Cancer Research Fund/American Institute of Cancer Research (World Cancer Research Fund International, 2018) recommendation to limit red meat to no more than 500 g per week. We directly used the World Cancer Research Fund/American Institute of Cancer Research cut‐off for red meat, because in the MDG, the recommended servings are for all animal products combined. We used the number of servings recommended in the MDG for legumes (two servings per day) and low fat dairy products (two servings per day; Bonvecchio Arenas et al., 2015). The MDG discourage the intake of foods high in sugar, fat, and energy density and highly processed foods, but the MDG do not give guidelines for specific amounts of these foods. We used an upper limit of 10% of energy intake as the recommendation for HSFAS products proposed by Batis, Aburto, Sanchez‐Pimienta, Pedraza, and Rivera (2016) for the Mexican healthy diet.

### 2.3 Birthweight

Offspring's birthweight was obtained from medical records. Birthweight (grams) was divided into three categories: <2,500 for low, 2,500–3,999 for normal, and >4,000 for high according to the WHO classification (WHO, 2004).

### 2.4 Potential confounders and intermediate variables

Maternal age, education, and number of pregnancies (parity) were obtained using questionnaires that collected data on socio‐demographic variables, obstetric history, and detailed information about the pregnancy, including gestational age at birth. Maternal education was grouped by completed or no completed basic school (≥9 years and <9 years). Parity was divided into three groups (nulliparous, 1–2, and ≥3). Marital status was divided into two groups (married/partnered and divorced/single). Maternal height was measured at the first visit by trained staff using standardized methods (Lohman technique). Pre‐pregnancy weight was self‐reported by participants. The pre‐pregnancy body mass index (pBMI) was calculated as pBMI = kg/m^2^ and was categorized into five groups: underweight, ≤18.5; normal, ≥18.5–24.9; overweight, 25–29.9; obesity 1, 30–34.9; and obesity 2, ≥35.

Maternal weight was measured at the first and consecutive visits by trained staff using standardized methods (Lohman technique). Rate of gestational weight gain (RGWG; kg per week) was calculated in second and third trimesters and over the whole pregnancy. We categorized RGWG according to whether Institute of Medicine (Rasmussen & Yaktine, 2009) recommendations were met (insufficient, adequate, and excessive) based on ranges of the mother's pBMI. Recommended weight gain in the second and third trimesters was based on the assumption that underweight, normal weight, overweight, and obese women should gain weight within the normal range of 0.44–0.58, 0.35–0.50, 0.23–0.33, and 0.17–0.27 kg per week, respectively.

### 2.5 Statistical analysis

Descriptive statistics were computed for socio‐demographic variables and maternal characteristics.

#### 2.5.1 Dietary patterns

Factor analysis (FA) was applied to the women's daily intake (percentage of energy contribution) of each of the 12 food groups in order to reduce the large amount of diet information obtained into a smaller set of independent (non‐correlated) factors (Yong & Pearce, 2013). These factors reduced the 12 food groups to a smaller number based on their similar variability and allowed identification of specific dietary patterns (Thompson et al., 2010; Tucker, 2010) to facilitate interpretation of results.

Percentage of energy contributed by each food group to daily total energy intake for each individual was obtained using the following formula: (Energy intake by food group * 100)/(Total energy intake). We used the percentage of variance explained by each factor and a screen plot to determine the number of factors (Yong & Pearce, 2013). Distinct dietary patterns were defined for the second and third trimesters and for both periods together, characterized by high loadings of specific food groups during these intervals. Food groups were considered to be descriptive of the “dietary pattern” if their factor loadings had a magnitude of 0.3 or greater (Knudsen et al., 2008; Varraso et al., 2012). The signs of the loadings show the direction of the correlation between each factor and food group (Yong & Pearce, 2013).

For each pregnant woman, a factor score in the respective dietary pattern was estimated, and then individual factor scores were divided into tertiles. FA allowed us to obtain dietary patterns by computing coefficients for each food group in the analysis; individual dietary pattern scores were calculated by multiplying these coefficients by the individual's consumption of the groups to provide a natural score for every participant (Crozier, Robinson, Godfrey, Cooper, & Inskip, 2009; Elstgeest, Mishra, & Dobson, 2012).

To evaluate the similarity of the factor loadings across trimesters in each dietary pattern for each woman, we estimated a coefficient of congruence (Lorenzo‐Seva & Ten Berge, 2006). This coefficient is an index of factor similarity and is estimated as $s^{\left.\sum x_{i}y_{i}\right|}\sqrt{\sum y_{i}^{2}\sum x_{i}^{2}}$, in which a value between 0.85 and 0.94 indicates that the factors are fairly similar, and a value higher than 0.95 indicates that the two factors can be considered equal (Batis et al., 2014; Lorenzo‐Seva & Ten Berge, 2006).

#### 2.5.2 Maternal diet quality score

For the MDQS construction, a value of 1 was assigned if the recommendation was met and 0 if the recommendation was not met for each of the seven individual recommendations: (a) PUFAS, (b) added sugars, (c) fruits and vegetables, (d) red meat, (e) low fat dairy products, (f) legumes, and (g) HSFAS. The scores for each recommendation were then summed with a maximum score of 7 if all recommendations were met and 0 if no recommendations were met. We defined the three following categories of adherence: low (0–2 points), medium (3–4 points), and high (≥5 points).

Differences of nutrient intakes and socio‐demographic and maternal characteristics across the intake patterns and categories of MDQS were compared using chi‐square and analysis of variance for categorical and continuous variables, respectively.

Multivariable linear and logistic regressions were used to assess the association between maternal diet (dietary patterns and MDQS) and the risk of having an LBW infant; models were adjusted for potential confounders (parity, baby's sex, mother's height and age, education, gestational age at the end of pregnancy, education level, and pBMI). We included the potential effect for RGWG in our models because it has been proposed as a potential intermediate variable in the association between maternal diet and fetal growth.

STATA (Stata for Mac 13.0, Drive East College; E.U) was used for statistical analysis.

## 3 RESULTS

### 3.1 Dietary patterns

Six hundred and sixty pregnant women from the cohort had complete information on key variables of interest for this study. Socio‐demographic and maternal characteristics according to birthweight classification are shown in Table 1. The proportion of babies who had LBW was 7.42% (*N* = 49). We found significant differences in GWGR (kg per week) comparing normal and LBW categories (0.381 ± 0.163 vs. 0.315 ± 0.171, *P* = .01). We identified a higher proportion of preterm births in babies with LBW compared with those babies who were born with normal weight (44.90 vs. 27%, *P* = .005).

**Table 1:** Socio‐demographic and maternal characteristics in a sample of 660 women from the PRINCESA cohort

Variable	Overall sample (*n* = 660)^a^	Normal birthweight (*n* = 611)^a^	Low birthweight (*n* = 49)^a^
Maternal height, cm (±*SD*)	156.05 (5.9)	156.01(5.9)	155.92 (5.1)
Maternal age, years (±*SD*)^c^^,^ d	25.08 (5.8)	25.16 (5.9)	24.10 (5.1)
Birthweight, g (±*SD*)^b^	3071.4 (442.7)	3129.22 (331.8)	2164.69 (261.9)
Baby sex, *n* (%)			
Females	297 (45.0)	277 (45.3)	20 (40.8)
Males	331(50.1)	303 (49.5)	28 (57.1)
Missing	32 (4.8)	31 (5.0)	1 (2.0)
Pre‐pregnancy BMI kg/m^2^ (±*SD*)	25.72 (5.2)	25.76 (5.1)	25.50 (5.8)
BMI classification (kg/m^2^), *n* (%)^c^			
<18.5	29 (4.3)	26 (4.3)	3 (6.2)
≥18.5 < 25	301 (45.6)	269 (44.6)	22 (45.8)
≥25 < 30	215 (32.5)	206 (34.1)	13 (27.0)
≥30 < 35	82 (12.4)	73 (12.1)	8 (16.6)
≥35	33 (5.0)	29 (4.8)	2 (4.1)
Gestational weight gain rate (kg per week) (±*SD*)	0.375 (0.1)	0.381 (0.1)	0.315 (0.1)
Gestational weight gain, *n* (%)^b^			
Insufficient	196 (29.7)	168 (28.8)	21 (42.8)
Adequate	194 (29.3)	169 (28.9)	19 (38.7)
Excessive	270 (40.9)	246 (42.2)	9 (18.3)
Term of gestation, *n* (%)^b^			
Preterm	65(9.8)	27 (7.0)	22 (44.9)
Term	595 (90.1)	568 (92.9)	27 (55.1)
Parity, *n* (%)^b^			
Nulliparous	317 (48.0)	288 (47.1)	29 (59.1)
1–2	188 (28.4)	174 (28.4)	14 (28.5)
≥3	154 (23.3)	148 (24.2)	6 (12.2)
Missing	1(0.001)	1 (0.16)	0 (0.0)
Marital status			
Single or divorced	170 (25.7)	157 (25.7)	13 (26.5)
Married/partnered	489 (74.0)	453 (74.1)	36 (73.4)
Missing	1 (0.001)	1 (0.16)	0 (0.0)
Maternal education, *n* (%)^c^			
≤9 years	370 (56.0)	345 (56.4)	25 (51.0)
>9 years	290 (43.9)	266 (43.5)	24 (48.9)

Abbreviation: BMI, body mass index.

a Values are *n* (%) or means ± *SD*s.

b Significantly different between categories of birthweight (low versus normal).

c Significantly different between categories of Maternal Diet Quality Score.

d An adequate gestational weight gain rate (kg per week was defined according to Institute of Medicine [IOM] recommendations).

Using FA on a total of 12 food groups, we identified two distinct dietary patterns in the second and third trimesters. The third and subsequent factors explained less variation than the first two and were less interpretable, so they were not considered further. Dietary pattern for whole pregnancy included the total number of 24H‐DR obtained during second and third trimesters of pregnancy. This pattern was used in the association models because the sum of report intake represents more accurately the usual food intake.

The first factor explained 12.89% and 13.03% of the variation in the dietary data for the second and third trimesters, respectively. In both trimesters, this factor was characterized by high intake of white meat and eggs, low fat dairy products, cereals, tubers, fruits, and vegetables and low intake of HSFAS, sugary drinks, juices, and sodas; we termed this the “healthier dietary pattern.”

Factor 2 explained between 11.83% and 11.22% of the variation in the second and third trimesters, respectively. It was characterized in the second trimester by high intakes of sugary drinks, juices and sodas, red and processed meat, cereals, and tubers. In the third trimester, it was characterized by high intakes of HSFAS, red and processed meat, and dairy products (both low and high fat); unlike in the second trimester, this factor was not characterized by a high intake of sugary drinks, juices, and sodas, so we termed it a “mixed dietary pattern.” We used the denomination of “mixed dietary patterns” in second and third trimesters because factors for both dietary patterns were generated sequentially with the same 12 food groups (Table S1). The low congruence between trimesters can be explained by increase in adherence to the “healthier dietary pattern.” It is important to mention that the sequential nature of the determination of factors implies the possibility that some women changed scores between trimesters.

Younger maternal age (24.25 ± 5.2 vs. 26.20 ± 6.2 years, *P* < .001) and nulliparity (35.64 vs. 29.33%, *P* < .08) were associated with lower adherence to healthier dietary pattern (tertile 1 vs. tertile 3). We did not observe significant differences with respect to other characteristics or across the tertiles of the mixed diet pattern.

The coefficient of congruence between trimesters 2 and 3 was very high (0.98) for the healthier dietary pattern and low (0.31) for the mixed dietary pattern. The factor loadings of the food groups from FA in the second and third trimesters and during the complete pregnancy are shown in Table 2.

**Table 2:** Factor loadings of food groups in derived dietary patterns of women participating in the PRINCESA cohort

Food group^a^		Trimester 2		Trimester 3		Whole pregnancy	
		Healthier dietary pattern	Mixed dietary pattern	Healthier dietary pattern	Mixed dietary pattern	Healthier dietary pattern	Mixed dietary pattern
1	Oils and fats	−0.03	−0.07	−0.06	0.09	−0.21	**−0.42**
2	HSFAS	**−0.50**	**−0.54**	**−0.48**	**0.45**	**−0.39**	**−0.35**
3	SSBs	**−0.39**	**0.45**	**−0.46**	**−0.72**	**−0.39**	**0.31**
4	Sugars and candies	−0.07	−0.17	−0.06	−0.08	−0.06	−0.11
5	Red and processed meat	−0.16	**0.67**	−0.18	0.24	−0.07	**0.65**
6	White meat and eggs	**0.43**	**−0.40**	**0.38**	**−0.48**	**0.44**	**−0.33**
7	Low fat dairy products	**0.43**	−0.06	**0.50**	**0.34**	**0.40**	**−0.35**
8	High fat dairy products	−0.06	**−0.33**	−0.01	0.29	−0.01	−0.02
9	Legumes	−0.23	0.01	−0.25	0.18	**−0.40**	0.02
10	Cereals and tubers	**0.49**	**0.30**	**0.40**	−0.21	**0.46**	**0.45**
11	Supplements	−0.04	0.24	−0.09	−0.04	−0.06	**0.30**
12	Fruits and vegetables	**0.64**	0.08	**0.65**	0.11	**0.71**	0.06
Eigenvalue		1.54	1.41	1.56	1.34	1.61	1.37
% explained variance		12.89	11.83	13.03	11.22	13.59	11.53

Abbreviations: CI, confidence interval; HSFAS, high saturated fat and/or added sugar foods; *SD*, standard deviation; SSBs, sugar‐sweetened beverages.

a Food groups were considered to be descriptive of the dietary pattern if their factor loadings had a magnitude of 0.3 or greater (values in bold).

On average, women with scores in the highest tertile of the two patterns reported less energy intake, higher intake of cereals and tubers, and lower intake of HSFAS (*P* < .05). Food groups with high positive factor loadings were highest in tertile 3, whereas food groups with high negative factor loadings were highest in tertile 1 for both dietary patterns. Food group intakes according to tertiles of dietary patterns are presented in Table S2.

### 3.2 Maternal Diet Quality Score

MDQS ranged from 0 (reflecting no adherence at all) to 7 (reflecting maximum adherence). On average, pregnant women with greater adherence (≥5 points) to MDQS were older (24.02 ± 5.23 vs. 25.83 ± 6.71 years; *P* < .05) and had the lowest proportion of total LBW (34.00% vs. 12.00%; *P* < .05) compared with women with lower adherence (<3 points). We did not observe significant differences in the other socio‐demographic characteristics across the categories of MDQS.

The mean (±*SD*) of MDQS was 3.06 (±1.25) for second trimester and 3.30 (±1.27) for third trimester and 3.09 (±1.26) for whole pregnancy (averaged over the last two trimesters).

A positive trend in MDQS values was seen from tertiles 1 to 3 of the healthier dietary pattern (2.81 ± 1.10, 3.52 ± 1.0, and 4.09 ± 1.13, *P* < .001). No trend was observed in the MDQS values from tertiles 1 to 3 of the mixed dietary pattern. The average intakes of different diet components and scores according to tertiles of dietary patterns and categories of MDQS are presented in Table S2.

We found that the maternal diet quality improved from the second trimester to the third trimester. In the second trimester, the percent of women with lower, medium, and higher adherence to MDQS were 33.84% (95% confidence interval [CI] [31.34, 36.32]), 53.91% (95% CI [51.28, 56.52]), and 12.26% (95% CI [10.53, 13.98]), respectively. For the third trimester, the percent of women with lower adherence dropped, and those with higher adherence increased (*P* < .001); the percentage distribution was as follows: 27.49% (95% CI [24.84, 30.15]), 56.55% (95% CI [53.6, 59.49]), and 15.94% (95% CI [13.77, 18.12]) in lower, medium, and higher adherence categories, respectively. Significance was <.001 by chi‐square test.

In accordance with the previous data, we also identified that intake of energy and some nutrients such as carbohydrates, total fats, SF, PUFAs, added sugars, fibre, and iron were higher (*P* < .005) in the second trimester compared with the third trimester. In contrast, intakes of folate and calcium were higher in the third trimester compared with the second trimester (*P* < .05).

The prevalence of excessive saturated fat consumption (>10% of total energy intake) was elevated, especially in the second trimester of pregnancy (53.04%; 95% CI [49.60, 57.31]). The prevalence of excessive added sugar intakes was 35.67% (95% CI [32.22, 39.09]) and 26.80% (95% CI [23.42, 30.19]) in the second and third trimesters, respectively. The inadequacy of fibre decreased from 55.11% (95% CI [51.28, 58.93]) to 53.72% (95% CI [49.90, 57.64]) between two last trimesters. Table S3 shows the energy and nutrients intakes of this sample at the second and third trimesters in our population.

Discretionary food groups (SSBs, HSFAS, sugar, and candies) contributed 36.60% of the total energy intake in the second trimester and 38.21% in the third trimester. Basic foods contributed 63.40% and 67.21% of the total energy intake of the sampled women in the second and third trimesters, respectively. Dairy products and legumes were among the groups with the lowest %EC in both trimesters.

Figure S1 shows the percentage contributions of each food group to total energy intake in the second and third trimesters. These observed differences in MDQS, nutrient intakes, and energy contribution of food groups between trimesters indicate that the diet composition of women is modified for the better as pregnancy progresses.

### 3.3 Models

Table 3 shows the crude and multivariate‐adjusted odds ratios (ORs) and (95% CIs) of having an LBW infant across tertiles of consumption of two dietary patterns. Compared with women in the lowest tertile of the mixed dietary pattern, those in the highest tertile had higher risk (OR, 1.58; 95% CI [0.63, 3.44]) of having an LBW infant. On the other hand, women in tertiles 2 and 3 of the healthier dietary pattern had the lowest risk (OR, 0.47; 95% CI, [0.23, 1.00] and OR, 0.81; 95% CI [0.35, 2.12]; tertile 1 as reference). However, these associations were not statistically significant, even after adjustment for potential confounding variables.

**Table 3:** Association between dietary patterns during pregnancy and risk of low birthweight

Adherence	Healthier dietary pattern				Mixed dietary pattern			
	β [95% CI]	*P*	OR [95% CI]	*P*	β [95% CI]	*P*	OR [95% CI]	*P*
Continuous score	Crude: −2.01 [−37.8, 33.8]	.91	Crude: 0.94 [0.70, 1.32]	.7	Crude: −7.08 [−41.3, 27.1]	.68	Crude: 1.12 [0.8, 1.4]	.42
	Adjusted^a^: −1.71 [−36.3, 32.9]	.92	Adjusted^a^: 0.85 [0.59, 1.23]	.41	Adjusted^a^: −1.01 [−33.6, 31.6]	.95	Adjusted^a^: 1.11 [0.76, 1.56]	.65
Low adherence (T1)	Reference		Reference		Reference		Reference	
Medium adherence	Crude: 7.29 [−0.7, 91.6]	.86	Crude: 0.81 [0.4, 1.6]	.58	Crude: 59.12 [−25.5, 143.8]	.90	Crude: 1.14 [0.55, 2.3]	.70
(T2)	Adjusted^a^: 11.48 [−68.7, 91.6]	.77	Adjusted^1^: 0.66 [0.2, 1.5]	.33	Adjusted^a^: 90.47 [8.7, 172.1]	.91	Adjusted^1^: 0.98 [0.4, 2.2]	.94
High adherence (T3)	Crude: 0.77 [−87.4, 87.6]	.99	Crude: 0.88 [0.4, 1.7]	.72	Crude: 117.06 [5.4, 228.6]	.98	Crude: 1.16 [0.5, 2.4]	.71
	Adjusted^a^: 6.23 [−77.3, 89.8]	.88	Adjusted^a^: 0.65 [0.2, 1.5]	.33	Adjusted^a^: 108.01 [0.48, 215.52]	.93	Adjusted^a^: 1.12 [0.4, 2.5]	.78

Abbreviations: CI, confidence interval; OR, odds ratio.

a Logistic models adjusted for energy intake (continuous), dietary patterns (healthier and mixed) were mutually adjusted, pre‐pregnancy BMI (normal, overweight, obesity 1, and obesity 2), parity (nulliparous, 1–2, and ≥3 pregnancies), gestational weight gain (insufficient, adequate, and excessive), maternal age (tertiles), maternal height (continuous), marital status (nonpartnered and married/partnered), maternal education (basic ≤ 9, superior > 9 years), term of gestation (preterm, term), and baby's sex (female, male).

On the other hand, higher adherence to MDQS (category 1 as reference) was associated with a reduced risk of having an LBW baby (OR, 0.60; 95% CI [0.46, 0.82], *P* < .05) for each increase of one standard deviation in the score. When examined by category, the highest adherence was associated with a reduced risk of LBW (OR, 0·34; 95% CI [0.11, 0.90], *P* < .05; Table 4) compared with the lowest adherence category (reference group).

**Table 4:** Association between MDQS during pregnancy and risk of low birthweight

Adherence to MDQS	β [95% CI]	*P*	OR [95% CI^a^]	*P*
Continuous score^a^	Crude: 28.17 [−5.48, 61.83]	.101	Crude: 0. 61 [0.50, 0.93]	.001^**^
	Adjusted^b^: 30.28 [−2.06, 62.90]	.004^**^	Adjusted^b^: 0.53 [0.46, 0.82]	<.001^**^
Low adherence	Reference		Reference	
Medium adherence	Crude: 59.12 [−25.57, 143.82]	.171	Crude: 0.46 [0.24, 0.87]	.017^**^
	Adjusted^b^: 90.47 [8.76, 172.17]	.030^**^	Adjusted^b^: 0. 36 [0.17, 0.75]	.006^**^
High adherence	Crude: 117.06 [5.44, 228.62]	.040^**^	Crude: 0. 26 [0.85, 0.79]	.018^**^
	Adjusted^b^: 108.01 [0.48, 215.52]	.049^**^	Adjusted^b^: 0.22 [0.06, 0.75]	.016^**^

Abbreviations: CI, confidence interval; OR, odds ratio.

a Increment per each *SD.*

b Logistic models adjusted for energy intake (continuous), pre‐pregnancy BMI (normal, overweight, obesity 1, and obesity 2), parity (nulliparous, 1–2, and ≥3 pregnancies), gestational weight gain (insufficient, adequate, and excessive), maternal age (tertiles), maternal height (continuous), marital status (nonpartnered, married/partnered), maternal education (basic ≤ 9 years, superior > 9 years), term of gestation (preterm, term), and baby's sex (female, male).

## 4 DISCUSSION

In this sample of Mexican pregnant women from the Pregnancy Research on Inflammation, Nutrition, & City Environment: Systematic Analyses cohort, we found a reduced risk of LBW in women who had high adherence to MDQS, in comparison with women with lower adherence. Our results are consistent with other cohort studies evaluating quality scores during pregnancy and infant birthweight. The Infancia y Medio Ambiente (Childhood and Environment) Mother and Child Cohort Study (INMA) reported that women with the highest quintile quality scores of Alternate Healthy Eating Index had a significantly lower risk of delivering a fetal growth‐restricted infant (OR, 0.24; 95% CI [0.10, 0.55]; *P* = .001) than women in the lowest quintile (Rodriguez‐Bernal et al., 2010). The INMA study also evaluated impact of MD adherence during pregnancy on fetal growth in 2,461 mother/new‐born pairs in Spain and Greece; women with high MD adherence had a significantly lower risk of delivering a fetal growth‐restricted infant (RR, 0.5; 95% CI [0.3, 0.9]). In a cohort of 862 pregnant women from New Hampshire recruited at 24–28 weeks of gestation, Emond et al found that increased diet quality appeared to be linearly associated with a reduced likelihood of small for gestational age (*P*‐trend = .03), although each quartile comparison did not reach statistical significance. Specifically, ORs for small for gestational age were 0.89 (95% CI [0.37, 2.15]), 0.73 (95% CI [0.28, 1.89]), and 0.35 (95% CI [0.11, 1.08]) for each increasing quartile of diet quality compared with the lowest quartile (Emond et al., 2018). The Australian Longitudinal Study on Women's Health reported that women with the highest Australian Recommended Food Score had the lowest odds of delivering an LBW child (OR = 0.4; 95% CI [0.2, 0.9]; Gresham, Collins, Mishra, Byles, & Hure, 2016).

On the other hand, The Growing Up in Singapore Towards Healthy Outcomes Study (Chia et al., 2018) and The Infant Feeding Practices Study II (Poon et al., 2013) did not find association between diet quality and birthweight or fetal growth. These inconsistent results between studies can be associated to the use of different cut‐offs for determining adherence to specific predefined dietary pattern. Our findings with respect to derived dietary patterns do not offer evidence that the healthier dietary pattern in this population protects against LBW, in contrast to what has been observed in other studies (Knudsen et al., 2008; Okubo et al., 2012; Thompson et al., 2010). Only one study conducted in the U.S. population had findings consistent with our results, Colón‐Ramos et al. (2015) examined data from the longitudinal cohort Conditions Affecting Neurocognitive Development and Learning in Early Childhood to explore the association between maternal dietary patterns and offspring size at birth (birthweight, length, and head circumference); seven dietary patterns were derived (Healthy, Healthy Processed, Healthy Southern, Mixed, Processed, Processed Southern, Southern) using exploratory FA (EFA) with varimax rotation method. Study reported that even after controlling for confounders, any dietary pattern was associated with birthweight in this population.

Although we observed an increase of risk of LBW in women with the highest consumption of the mixed dietary pattern, results were not statistically significant even after controlling for potential confounders or intermediate variables including gestational weight gain. A potential explanation for these results could be the antagonistic interaction among beneficial and harmful food groups in the mixed dietary pattern, for example, cereals, supplements, and SSBs.

Another possible cause for these inconsistent findings may be due to the methods used to derive dietary patterns. PCA is most commonly used for dietary pattern analysis; however, we used EFA because it takes common variance in observed variables into account whereas PCA only considers total variance. When defining patterns, subjective decisions are introduced at various points, such as decisions for cut‐offs for food‐group loadings or type/need of rotation. Furthermore, the eigen value >1, screen plot, and interpretability are mostly used to determine the number of patterns to retain. Factors rotation can maximize the variability among the loadings in each factor however, we did not find differences after rotation. In addition, EFA allows to identify new dietary pattern variables that are obtained from underlying interrelationships between the dietary components and usually capture other lifestyle and socio‐economic conditions related to diet (Hodge & Bassett, 2016); on the other hand, a priori defined dietary patterns tended to show stronger associations with the outcomes than individual food groups (Ocké, 2013).

In relation to consumption of discretionary food groups (sugary drinks, juices, sodas, HSFAS products, sugar, and candies), we observed that contribution of the total energy intake was lower in the third trimester than in the second trimester. We also identified that intakes of energy, carbohydrates, total fats, SF, and added sugars were higher in the second trimester compared with the third trimester. With respect to diet quality, the proportion of women with high adherence to MDQS was greater in the third trimester than in the second trimester. However, we identified that the healthier dietary pattern was very similar between trimesters 2 and 3, but the mixed dietary pattern was not.

Only one study has explored dietary intake changes during pregnancy among 12,572 women in the United Kingdom; the results support that dietary patterns are similar throughout pregnancy, but diet composition quality is modified for the better as pregnancy progresses (Crozier et al., 2009), as observed in our study.

The principal strengths of the present study include the prospective design that provided a valuable opportunity to assess dietary differences in the second and third trimesters for the first time in low‐income urban women. Our cohort included women with uncomplicated pregnancies, and thus, our findings could be relevant to a wide population of women worldwide. The results on diet composition and characterization of dietary patterns also provide insights into which foods are more accessible in this population context.

The high consumption of unhealthy food groups (HSFAS and SSBs) reported by these women could be explained by the lower prices and high market availability for this type of food, whereas the lower consumption of fruits, vegetables, legumes, and other traditional foods may be due to higher prices and low salaries (CONEVAL, 2015) that make it difficult for pregnant women to access healthy foods and high quality diets.

To our knowledge, this is the first study to report the characterization of maternal diet during pregnancy using two approaches: a posteriori (FA) and a priori (MDQS). In Mexico, no published studies to date have evaluated dietary patterns or diet quality in pregnant women. Considering that there is currently not one best approach to study overall diet, the use of these complementary approaches that include classification of diet patterns by selected nutriments and foods (a priori) and dietary patterns derived from specific populations (a posteriori) may be useful for identification of food groups that may contribute to pregnant women health. The present work provides valuable new knowledge on areas of opportunity to improve the quality, dietary patterns, and composition of the diet in pregnant women who live in similar contexts. This methodology for diet analysis allowed identification of foods which are part of the usual dietary pattern in Mexican pregnant women; on the other hand, a priori defined dietary patterns (MDQS) showed a stronger association between Mexican recommendations and LBW, so it could be considered as a useful tool to test if current dietary recommendations have a measurable protective effect against LWB in other contexts and different perinatal outcomes.

Some limitations exist. Although dietary intake was assessed monthly and intakes in the previous 4 weeks were recorded, food intake measures at only one point without repeated measures in consecutive days do not capture the day‐to‐day variability in dietary intake and therefore do not allow construction of the distribution of usual intake.

As in any dietary study, recall bias could have occurred. Other biases related to dietary pattern derivation are connected with limitations related to the reference database of food composition that we used (diversity in food preparation, various sources of information, and subjectivity in select serving sizes and serving grouping) as well as the FA method (nature of the factor scores, subjectivity in the manner in which foods are grouped, data treatment, rotation decision, plausible limits, and validity). Gestational weight gain is a mediating factor that may lead to underestimation of its association with LBW when introduced as an intermediate variable. Several underlying factors such as oxidative stress and systemic inflammation must be explored in complementary studies in order to understand mediating pathways for LBW.

In summary, our findings provide evidence that higher adherence to MDQS is associated with lower risk of having a LBW baby. Our results are important for design and implementation of policy‐based health prevention programs because they support the role of a healthy overall diet in preventing negative pregnancy outcomes such as LBW rather than promoting individual nutrient supplements or avoidance of individual foods or nutrients. This represents a more comprehensive and complementary approach to public health.

Eating a healthy diet during pregnancy is crucial for the future health of the unborn child and future generations; thus, all pregnant women should be encouraged to eat a healthier dietary pattern and high quality diet, using dietary recommendations that are simple, accessible, and well suited to the population context.

Further investigation of these findings, by replicating this methodology in different regions to identify social and environmental aspects related to accessibility of a better quality diet, is warranted.

## CONFLICTS OF INTEREST

The authors declare that they have no conflicts of interest.

## CONTRIBUTIONS

MSO, FVO, BS, and MAM designed research; MSO, FVO, MCC, and MAM conducted research, MAM, MSO, FVO, CBR, and SRR analysed data, and MAM, JARD, MSO, and FVO wrote the paper. MAM, MSO, and FVO had primary responsibility for final content. All authors read and approved the final manuscript.

## Supporting information

Table S1. Description of food groupings used to derive dietary patterns.
Table S2. Energy intake and food group consumption according to adherence to the dietary patterns and Maternal Diet Quality Score (MDQS).
Table S3. Energy, nutrients and prevalence of inadequate intakes in second and third trimesters of pregnancy.
Figure S1. Energy contribution of food groups in second and third trimesters.
Figure S2. Energy contribution of food groups in second and third trimesters.
