# Supplementary material for: Dietary patterns and diet quality during pregnancy and low birthweight: The PRINCESA cohort
Source: Matern Child Nutr. 2020 Feb 9;16(3):e12972. doi: 10.1111/mcn.12972 (PMC7296796; doi:10.1111/mcn.12972)
Supplement: Supplementary file 1 — Table S1. Description of food groupings used to derive dietary patterns. Table S2. Energy intake and food group consumption according to adherence to the dietary patterns and Maternal Diet Quality Score (MDQS). Table S3. Energy, nutrients and prevalence of inadequate intakes in second and third trimesters of pregnancy. Figure S1. Energy contribution of food groups in second and third trimesters. Figure S2. Energy contribution of food groups in second and third trimesters. [file MCN-16-e12972-s001.docx]

**Supplementary data**

**Supplemental Table 1. Description of food groupings used to derive dietary patterns.**

| Food group name | Food items |
| --- | --- |
| Oils and fats | Vegetable oils (e.g. olive oil) margarine, avocado and guacamole. |
| Foods high in saturated fat and/or added sugar (HSFAS) | Fast food (i.e. hot dog, pizza, hamburger, French fries), sweet bread, sweet cereals, cakes, cookies, pies, pastries, frozen dairy dessert, pudding, high fat Mexican antojitos (i.e. tamal, pambazo, fried tacos/quesadillas), fried snacks. |
| Sugar-sweetened beverages (SSBs) | Beverages with added sugars (sodas; fruit, flavored, sports, and energy drinks; aguas frescas (a traditional Mexican beverage usually prepared with water, fruit, and table sugar) and homemade SSBs; coffee and tea; and sweetened milk) |
| Sugars and candies | Sugar table, sweet sauces, candy (chocolate and non-chocolate), fruits in syrup, candied fruits, honey, jam, jelly, preserves, sugar, frosting and glaze syrup. |
| Red and processed meat | Beef, pork (fresh and cured), lamb, organ meats, cold cuts and sausages. |
| White meat and eggs | Poultry, fish, shellfish, seafood, eggs. |
| Low fat dairy products | Low fat milk and yogurt, white cheese (e.g. panela) |
| High fat dairy products | Whole milk, high fat yogurt, yellow cheese (i.e. cheddar, american, mozzarella) |
| Legumes and nuts | Beans, soy, chickpeas, lentil, seeds, nuts. |
| Cereals and tubers | Tortilla, salad bread, pasta, high fiber ready-to-eat cereals (e.g. All Bran), non-fried corn products. |
| Supplements | Milk/whey protein supplements, Ensure, Glucerna. |
| Fruits and vegetables | Banana, orange, grapes, melon, guava, mango, mandarin and all fresh fruits.  Carrots, tomatoes, dark green leafy vegetables, onion, broccoli, zucchini and fresh/frozen vegetables. |

**Supplemental Table 2. Energy intake and food group consumption according to adherence to the dietary patterns and Maternal Diet Quality Score (MDQS).**

| **Variable** | **Healthier dietary pattern** | | | | **Mixed dietary pattern** | | | | **MDQS** | | | |  |
| --- | --- | --- | --- | --- | --- | --- | --- | --- | --- | --- | --- | --- | --- |
|  | **Low** | **Medium** | **High** | **P*** | **Low** | **Medium** | **High** | **P*** | **Low** | **Medium** | **High** | **P*** | |
| MDQS | 2.81 (1.10) | 3.52 (1.01) | 4.09 (1.13) | <0.001* | 3.46 (1.1) | 3.33 (1.1) | 3.32 (1.2) | 0.41 | 1.65 (0.5) | 3.42 (0.5) | 5.24 (0.4) | <0.001** | |
| Energy, kcal (±SD) | 2385.09 (574.5) | 2089.02 (533.8) | 1899.63 (519.8) | <0.001* | 2242.08 (644.24) | 2117.01 (558.72) | 2014.59 (613.37) | <0.001* | 2140.61 (609.61) | 2106.37 (608.71) | 2180.69 (639.62) | 0.51 | |
| **Food groups** |  | | | | | | | | | | | | |
| Oils and fats, % (±SD) | 9.61 (9.01) | 8.06 (7.92) | 5.09 (6.13) | <0.001* | 11.81 (10.3) | 6.96 (6.2) | 3.72 (3.7) | <0.001* |  |  |  |  | |
| HSFAS, %(±SD) | 21.02 (9.57) | 18.18 (8.66) | 13.32 (7.63) | <0.001* | 20.81 (20.3) | 18.39 (7.9) | 13.328 (7.4) | <0.001* | 21.51 (8.48) | 18.16 (7.91) | 10.22 (9.27) | <0.001** | |
| SSBs, %(±SD) | 20.52 (9.69) | 16.51 (7.71) | 12.49 (7.22) | <0.001* | 12.79 (7.10) | 17.70 (7.73) | 19.12 (10.11) | <0.001* |  |  |  |  | |
| Sugars and candies, %(±SD) | 1.42 (3.21) | 1.11 (2.35) | 0.88 (2.51) | 0.18 | 1.58 (3.4) | 1.11 (2.4) | 0.82 (2.1) | <0.001* |  |  |  |  | |
| Red and processed meat, %(±SD) | 6.32 (4.36) | 5.95 (4.71) | 5..51 (4.53) | 0.20 | 3.42 (2.42) | 4.79 (2.61) | 9.70 (5.10) | <0.001* | 7.50 (4.91) | 5.52 (4.39) | 5.51 (3.91) | <0.001** | |
| White meat and eggs, %(±SD) | 4.51 (3.23) | 6.66 (4.14) | 9.38 (6.21) | <0.001* | 9.01 (6.40) | 6.42 (4.07) | 5.15 (3.75) | <0.001* |  |  |  |  | |
| Low fat dairy products, %(±SD) | 1.22(1.71) | 1.9(2.21) | 3.61 (3.60) | <0.001* | 3.62 (3.68) | 1.82 (2.16) | 1.4 1 (1.00) | <0.001* | 1.91 (2.10) | 2.13 (2.86) | 3.20 (3.61) | <0.001** | |
| High fat dairy products, %(±SD) | 5.23 (4.47) | 5.91 (4.32) | 5.23(5.01) | 0.17 | 5.3 (4.7) | 5.9 (4.4) | 5.1 (4.6) | 0.16 |  |  |  |  | |
| Legumes, %(±SD) | 7.65 (7.36) | 4.15 (5.31) | 2.29 (3.71) | <0.001* | 4.06 (5.2) | 5.03 (6.1) | 4.86 (6.6) | 0.17 | 3.42 (5.01) | 4.91 (63.27) | 5.39(6.31) | <0.05** | |
| Cereals and tubers, %(±SD) | 12.52 (4.51) | 15.81 (5.83) | 20.19 (8.21) | <0.001* | 12.66 (4.72) | 16.01 (5.83) | 19.89 (8.29) | <0.001* |  |  |  |  | |
| Supplements, %(±SD) | 0.14 (0.9) | 0.05 (0.46) | 0.02 (0.24) | 0.60 | 0.07 (0.08) | 0.07 (0.08) | 0.15 (1.0) | 0.02 |  |  |  |  | |
| Fruits and vegetables, %(±SD) | 9.8 3(3.9) | 15.12 (5.03) | 21.37 (7.42) | <0.001* | 14.82 (7.93) | 15.41 (6.61) | 16.03 (6.61) | 0.25 | 12.45 (6.62) | 15.77 (7.45) | 18.49 (6.51) | <0.001** | |
| Polyunsaturated Fats, %(±SD) | 11.62 (2.96) | 10.61 (2.61) | 9.63 (2.5) | <0.001 | (3.47) | 10.41 (2.61) | 10.19 (2.37) | <0.001 | 6.62 (3.39) | 8.83 (4.37) | 10.68 (4.63) | <0.001** | |
| Added sugars, %(±SD) | 11.23 (6.13) | 9.29 (5.28) | 6.73 (3.78) | <0.001 | 8.42 (4.37) | 9.93 (6.12) | 8.77 (5.74) | <0.001 | 12.92 (5.43) | 8.51 (5.31) | 6.21 (3.47) | <0.001** | |

Abbreviations: SD, standard deviation; CI, confidence interval; HSFAS, high saturated fat and/or added sugar foods; SSBs, sugar-sweetened beverages.

*Data are presented as percentages (%) of the total energy intake and ±SD.

** P values are from ANOVAs for means, and chi-square for frequencies. Significance level <0.05.

**Supplemental Table 3. Energy, nutrients and prevalence of inadequate intakes in second and third trimesters of pregnancy.**

| **Nutrient intakes*** | **Trimester 2** | **Trimester 3** | **Whole pregnancy** |
| --- | --- | --- | --- |
| Total energy, kcal (±SD) ^a^ | 2413.0 (787.1) | 2319.7(803.0) | 2332.8(638.2) |
| Protein |  | | |
| Grams (±SD) | 99.8(36.2) | 100.3(38.2) | 97.7(28.0) |
| Percentage total energy (±SD) | 17.0(4.88) | 17.8(5.22) | 17.1(3.65) |
| Carbohydrate |  |  |  |
| Grams (±SD) ^a^ | 277.1(109.2) | 258.8(95.1) | 265.1(82.6) |
| Percentage total energy (±SD) | 45.9(9.2) | 45.4(9.6) | 45.5(7.1) |
| Total fats |  | | |
| Grams (±SD) ^a^ | 86.6(43.6) | 84.3(51.8) | 84.6(35.4) |
| Percentage total energy (±SD) | 31.7(9.1) | 31.2(10.1) | 31.9(7.4) |
| Saturated Fats |  |  |  |
| Grams (±SD) ^a^ | 28.5(13.9) | 27.7(17.5) | 27.8(11.2) |
| Percentage total energy (±SD) ^a^ | 10.6(3.5) | 10.5(4.1) | 10.7(2.0) |
| High intake (more than WHO recommendation), n (% ) ^a,b^ | 353(53.0) | 332(50.3) | 354(53.6) |
| Polyunsaturated Fats |  |  |  |
| Grams (±SD) | 23.9(22.2) | 23.4(23.2) | 23.4(16.0) |
| Percentage total energy (±SD) | 8.5(5.60) | 8.3(5.5) | 8.6(4.5) |
| Low intake (less than AI), n(%) ^a,c^ | 410 (62.18) | 423(64.1) | 350 (53.0) |
| Added sugars |  |  |  |
| Grams (±SD) ^a^ | 61.4 (59.3) | 48.0 (49.5) | 55.0(41.9) |
| Percentage total energy (±SD) ^a^ | 9.6 (7.0) | 7.9 (6.4) | 9.1 (5.5) |
| High intake (more than WHO recommendation), n(% ) ^a,b^ | 237(35.6) | 177(26.8) | 218(33.1) |
| Fiber |  |  |  |
| Grams (±SD) | 31.48 (18.4) | 30.86(16.7) | 29.9(12.7) |
| Lower intake (less than AI), n(%) ^a,d^ | 364 (55.1) | 355(53.7) | 312(47.2) |
| Critical micronutrients |  |  |  |
| Calcium |  |  |  |
| Miligrams (±SD) ^a, e^ | 1064.63(426.9) | 1083.21(401.4) | 1062.12(328.5) |
| Lower intake (less than EAR),n(%) ^a,e^ | 318(48.6) | 306(46.3) | 312(47.3) |
| Iron |  |  |  |
| Miligrams (±SD) ^a^ | 28.1(13.5) | 27.2(14.0) | 27.1(10.3) |
| Lower intake (less than EAR),n(%) ^a, f^ | 363(55.0) | 386(58.4) | 364(51.5) |
| Folate |  |  |  |
| Micrograms (±SD) ^a, g^ | 288.6 (137.5) | 298 (150.0) | 284.2(103.7) |
| Lower intake (less than EAR),n(%) ^a,g^ | 643(97.4) | 632(95.7) | 652(98.8) |

*Data are presented as mean±SD. Inadequacies are presented as prevalences.

^a^ Significant differences between trimesters (p<0.05). Values are from ANOVAs for means, and chi-square for frequencies.

^b^ An excessive intake of added sugars and saturated fat was defined as intake >10% of the total energy intake, according to WHO recommendations (WHO. Diet, nutrition and the prevention of chronic diseases. Report of a Joint WHO/FAO Expert Consultation. Geneva: World Health Organization; 2003. WHO Tech Rep Ser. 2008.).

^c^ A lower intake(AI) of PUFAs was defined as intake >=6% and <=11% of the total energy intake, WHO recommendations.

^d^ A lower intake (AI) of fiber was defined as intake <28 g, according to Institute of Medicine (IOM) recommendations (Dietary Reference Intakes for Energy, Carbohydrate, Fiber, Fat, Fatty Acids, Cholesterol, Protein, and Amino Acids (Macronutrients) [Internet]. Washington, D.C.: National Academies Press; 2005).

^e^ A lower intake(AI) of calcium was defined as intake <1000 μg, according to Institute of Medicine(IOM) recommendations.

^f^ A lower intake(EAR) of iron was defined as intake <27 mg, according to Institute of Medicine(IOM) recommendations.

^g^. A lower intake(AI) of folate was defined as intake <600 μg, according to Institute of Medicine(IOM) recommendations.

**Supplemental Figure 1. Energy contribution of food groups in second and third trimesters.**

^a^ HSFAS: High in saturated fat and/or added sugar products.

^b^ SSBs: Sugar-sweetened beverages.

**Supplemental Figure 2. Energy contribution of food groups in second and third trimesters.**

^a^ HSFAS: High in saturated fat and/or added sugar products.

^b^ SSBs: Sugar-sweetened beverages.
